# The Deep Learning Solutions on Lossless Compression Methods for Alleviating Data Load on IoT Nodes in Smart Cities

**DOI:** 10.3390/s21124223

**Published:** 2021-06-20

**Authors:** Ammar Nasif, Zulaiha Ali Othman, Nor Samsiah Sani

**Affiliations:** Center for Artificial Intelligence Technology (CAIT), Faculty of Information Science & Technology, University Kebangsaan Malaysia, Bangi 43600, Malaysia; zao@ukm.edu.my (Z.A.O.); norsamsiahsani@ukm.edu.my (N.S.S.)

**Keywords:** compression, inflation, data traffic, deep learning, internet of thing, IoT, memory, network, population, problem, pruning, smart city, compression method, pooling, entropy coding, dictionary coding, IoT market

## Abstract

Networking is crucial for smart city projects nowadays, as it offers an environment where people and things are connected. This paper presents a chronology of factors on the development of smart cities, including IoT technologies as network infrastructure. Increasing IoT nodes leads to increasing data flow, which is a potential source of failure for IoT networks. The biggest challenge of IoT networks is that the IoT may have insufficient memory to handle all transaction data within the IoT network. We aim in this paper to propose a potential compression method for reducing IoT network data traffic. Therefore, we investigate various lossless compression algorithms, such as entropy or dictionary-based algorithms, and general compression methods to determine which algorithm or method adheres to the IoT specifications. Furthermore, this study conducts compression experiments using entropy (Huffman, Adaptive Huffman) and Dictionary (LZ77, LZ78) as well as five different types of datasets of the IoT data traffic. Though the above algorithms can alleviate the IoT data traffic, adaptive Huffman gave the best compression algorithm. Therefore, in this paper, we aim to propose a conceptual compression method for IoT data traffic by improving an adaptive Huffman based on deep learning concepts using weights, pruning, and pooling in the neural network. The proposed algorithm is believed to obtain a better compression ratio. Additionally, in this paper, we also discuss the challenges of applying the proposed algorithm to IoT data compression due to the limitations of IoT memory and IoT processor, which later it can be implemented in IoT networks.

## 1. Introduction

The UN reported that by 2030, almost 60% of the world’s population will reside in big cities with almost 38 million residents, such as Tokyo followed by Delhi, Shanghai, Mexico City, São Paulo, and Mumbai, which are all ranked amongst the world’s most populated cities [1]. In 2014, there were 28 mega-cities with thrice the population than back in 1990, and this number was estimated to exceed 41 cities in 2030. In the European Union the urban population is expected to reach 80% in 2050. Now, more than 50% of the world’s population live in urban areas, where they consume 75% of the energy, and they are also responsible for 80% of the greenhouse effect [2]. In 2050 it is predicted that the largest 200 cities in the world will each have a minimum population of 3 million people and that Mumbai (Bombay) in India, for example, may exceed 42 million [3]. The cities’ infrastructure has been developed to cater to the demands of the new urban population. In the beginning, when wireless technologies had not been introduced yet, governments tried to connect buildings through cables and wires, and the cities containing these buildings have been referred to as wired cities [4]. Later the term “virtual cities” was proposed in order to show digital representations and manifestations as an infrastructure [5]. Consequently, many other new names emerged with different purposes, such as cyber city (virtual 3D and GIS) [6], digital city (web-based representative) [7], intelligent city (high capacity for learning and innovation) [8], sentient city (the experience of living in a city that can remember-correlate-anticipate) [9], sustainable city (reducing CO_2_ emissions with efficient energy), [10] and green city (reducing greenhouse gas emissions and pollution with minimizing waste and inefficient use of natural resources, along with maintaining biodiversity) [11]. Researchers in [12,13,14,15,16,17,18,19,20,21,22,23,24,25,26,27,28] tried to summarize the impacting factors which affected the development of smart cities.

**Figure 1:**
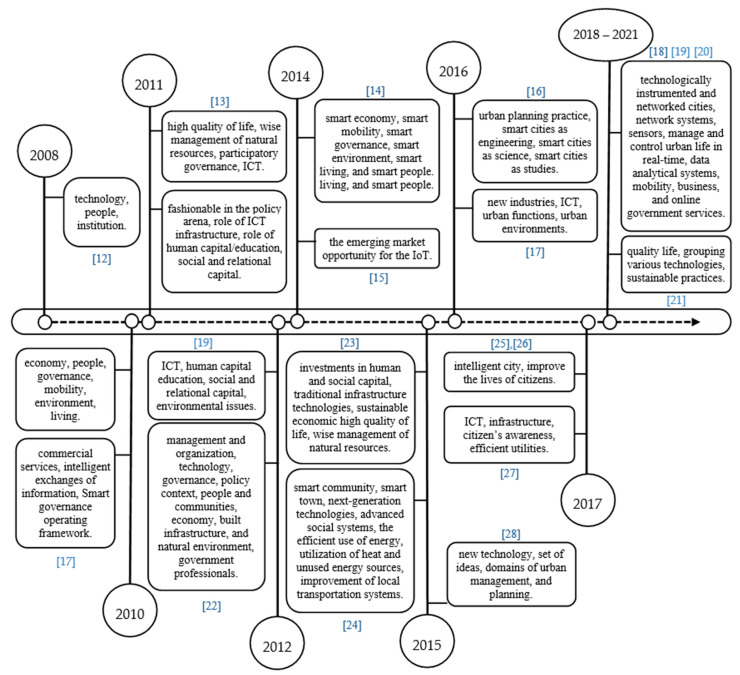
Chronology of factors on the development of smart cities.

Many efforts were made in order to satisfy the abnormal needs and requirements emerging from these urbanization movements, especially from the traditional management systems that provide service to billions of people, which was a nightmare for any government. Therefore, the concept of smart cities was introduced as a reliable solution for governments. The first smart city concept was introduced in the late 1990s [29]. At that time, most researchers defined a smart city as an urban area where data could be collected using various forms using electronic sensors connected to the internet. The information collected was then used in order to effectively control resources and services, and later used to optimize activities around the city. Consequently, many smart city definitions were proposed due to the various factors that influenced a smart city. These factors led to the change of smart city definition and affected its concept, as well. Figure 1 shows the chronology of the effecting factors that developed the various smart city definitions. All previous definitions have a common concept that the smart cities focus on the quality of life by using the latest technology and by offering new industries in order to promote urban development through many intelligent services systems. The IoT networks emerged on the market in 2014 taking on the form of infrastructure. Although the demand for IoT started in 2010, smart cities completely depended on networking systems and sensors, even in 2018, the IoT networks were in a high demand because it enabled analyzing data in real-time systems.

Nowadays, the notion of a smart city is globally used, and the number of smart cities has gradually increased. In 2012, approximately 143 smart cities, including 35 in North America and 47 in Europe, integrated new technologies into urban problem management [30]. Until now, smart city projects have increased in response to urbanization requirements and, as a result of the emerging technology, created capable infrastructures that can be used for new services. One of the most reliable technologies, which is considered the backbone of smart cities, is the IoT network because of its many features that fulfill the criteria needed for various smart city applications. Furthermore, it has a low cost compared to other traditional networks. All of the IoT components work as a single integrated system. The network has characteristics such as automation, intelligence, dynamicity, and zero-configuration, as illustrated in [31]. However, these IoT specifications still have limitations such as limited memory and low power processors. In smart cities with millions of people, most individuals own wearable devices and make use of IoT in their daily lives [32], such as smart homes and cars; therefore, heavy transmission of data every second over IoT networks is expected.

Furthermore, sometimes problems occur, especially when transmitting large amounts of data, such as delays in responding to citizens, which is highly expected in these large cities. This will be further discussed in the IoT memory challenge in section two, wherein it will be shown increasing the speed of data transfer, as a solo solution is not enough.

Later, many new technologies emerged, utilizing artificial intelligence, such as machine learning and deep learning [33], with the introduction of IoT as an infrastructure for smart cities [34]. This allowed edge computing to be implemented in the real world to alleviate the load on servers and sustain the implementation of smart cities. However, even during the 4th industrial revolution, several IoT applications still operate in a centralized structure [13]. Therefore, many researchers have tried to demonstrate the significance of IoT in applying edge computing and how it can be more feasible for smart cities [35]. Nevertheless, manufacturers and researchers were not sufficiently interested in developing IoT, particularly its limited memory capacity.

Memory size can be considered a critical problem in the IoT network because the small available memory segments messages into many smaller packets that require more transmission time, leading to consumption of more power and more latency [36]. A realistic example of this was stated in [37] where the RootMetrics smart city project relied on the IoT network as an infrastructure, and the enormous network data traffic caused system failure because the tiny IoT memory was unable to handle such massive data without intelligent management. It has been shown that when sensed data is sent directly to a gateway or server, it not only consumes excessive power but also increases the chance of data loss [38].

As a solution, many previous research studies have focused on enhancing the transmission range and speed. Scratchpad Memory (SPM) & Non-Volatile Memory Express (NVMe) memory types were developed in order to hold small items of data for rapid retrieval in IoT devices [39]. SPMs are software-controlled and require additional programmer effort [40], while NVMe enables the code to be executed directly. No code has to be copied to the Random Access Memory (RAM), which will reduce the boot-up time as well [21]. SPMs & NVMe were expensive enough to be implemented for IoT.

The key contributions of this study are summarized as follows:

(1) We study the technical side of IoT memory to clarify why small IoT memory cannot handle massive amounts of data.
(2) We investigate lossless compression algorithms as well as previous and current related work that has been used to reduce data size and illustrated detailed differences between them to clarify which can be used for IoT.
(3) We demonstrate the fundamentals of deep learning, which later help us understand the techniques used for dimension reduction and how we can use them to compress data in IoT memory.
(4) We implement experiments on five datasets using lossless compression algorithms to justify which fits better for IoT and which is more suitable for numeric and time series data type as IoT data type.

The paper is organized as follows: we investigate the technical details about IoT memory and why the small IoT memory cannot handle large data traffic, as well as how previous studies have tried to manage such large data using compression algorithms in Section 2. Then we investigate in more details the compression algorithms and methods in Section 3 and review algorithms that can be applied for numeric and time series data because of their similar characteristics with IoT data. In Section 4, deep learning fundamentals are illustrated in order to understand the techniques used for dimensionality reduction. We also investigate the current compression algorithms using deep learning in order to assess whether they, as well as traditional compression algorithms, can be used to compress the IoT data. However, we found that compression algorithms in deep learning do not share a similar concept with traditional compression algorithms. Additionally, we also discuss the potential of combining pruning and pooling in deep learning techniques with any suitable traditional compression algorithms. This paper describes how to minimize or compress the data to fit into a memory of IoT node in order to alleviate IoT data traffic in the IoT network.

## 2. Internet of Things

**Figure 2:**
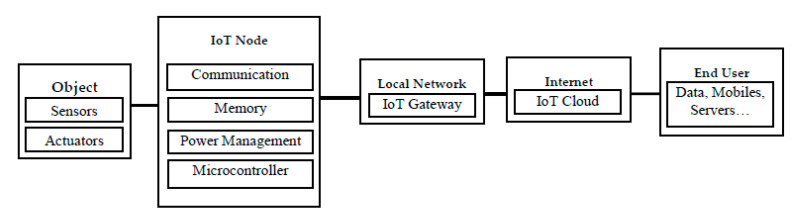
The IoT network architecture.

To illustrate in detail how an IoT system works, Figure 2 shows the IoT network architecture where every IoT node can be connected at least with one sensor or actuator or both. The node contains many integrated modules such as a processing unit (microcontroller), power management, memory (SRAM, Flash Memory, EEPROM), and communication modules (Wi-Fi, Bluetooth, 802.15.4 Wireless, wired). IoT nodes can be connected to an IoT gateway forming a local network. The gateway is connected to the internet which allows end-users to access (monitor or control) things.

### 2.1. IoT Memory

Memory is an essential component of an IoT device, as it stores both received and sent data. However, the performance of this memory depends on its type. One of these types is non-volatile memory (NVM), which retains data even if power is removed. The other type is volatile memory (VM), which loses data if power is removed. VM is faster than NVM but more expensive. Manufacturers using NVM for embedded devices have two options: one-time programmable (OTP) and multiple-time programmable (MTP). MTP offers applications that require long battery life, it is considered better than external flash memory and also lower in cost per bit. OTP is more suitable when the contents of memory cannot be modified once configured.

For IoT devices, manufacturers have developed scratchpad memories (SPMs) that are high-speed internal memories used for the temporary storage of calculations, data, and other works in progress. Ratzke stated in [39] that SPM is used to hold small items of data for rapid retrieval in IoT devices. In [40], researchers stated that SPM is different from cache memory because cache memory is managed by hardware while SPM is managed by software and requires additional effort from programmers. However, many researchers have focused on improving the IoT network by improving SPMs for performance gain, instead of focusing on data allocation, they focused on instruction allocation because IoT has embedded systems that have particular and special uses [39]. The researchers mentioned in [39] discovered that dynamic allocation of memory is better than static; therefore, there is no need to fill the memory before execution; instead, the memory should be filled when needed. Therefore, they proposed an algorithm that would decide whether to store memory objects (variables and code segments) in the SPM first or to the main memory before computing the addresses in the SPM. The SPM includes an array of SRAM cells and is used as an alternative to cache due to its specifications in energy efficiency, time predictability, and scalability. However, there is a need for the compiler or the programmer to allocate appropriate data to the SPM efficiently. Therefore, data management is the most challenging issue in systems equipped with SPMs, as researchers have stated in [41]. Furthermore, Lipman suggested one of the other ways to improve IoT devices would be using non-volatile memory (NVM). NVM is fast enough to allow executing the code directly, and there is no need to copy the code to the RAM here, which would reduce the boot-up time as well. However, there are still many improvements to be made, such as those in size and cost [21]. Because of this, manufacturers still use the traditional memory, which is the SRAM, to store data in IoT devices.

### 2.2. The IoT Memory Challenge

IoT memory has a low capacity, which is used in caching enormous network data, the IoT insufficient memory space is a crucial problem for smart city projects that rely on IoT networks as infrastructure. However, manufacturers of IoT devices have focused on increasing the speed of accessing data by proposing SPMs and NVM, as illustrated in the IoT memory section. Furthermore, they have focused on increasing the range of connections with low power consumption. Unfortunately, only a handful of researchers were interested in increasing the memory size both because the process was expensive and because this was not a critical issue since data was not large in the past.

**Figure 3:**
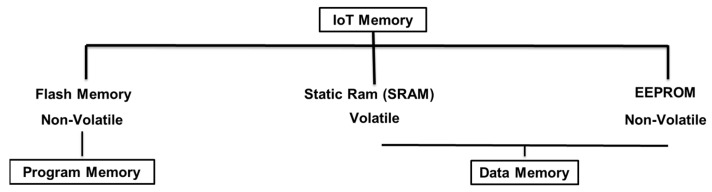
Three memory types for the IoT.

For more clarification, Figure 3 shows that IoT memories are of three types: non-volatile flash memory, which is used for programs, also known as program memory, and the other two types are for data and are known as data memory. A non-volatile EEPROM and volatile SRAM are used to temporarily store data. Memory sizes differ by controller type and version; the data that is received and transmitted through the network is stored in the SRAM. Data for Wi-Fi credentials, such as usernames and passwords, is stored in the EEPROM.

One of the challenges faced here is the insufficient memory size that causes buffer overflow, which can happen when software writes data to a buffer and anomalously overflows the capacity of the buffer, resulting in the overriding of adjacent memory positions. Information is transmitted into a container with insufficient space, and this information is then replaced by the data in neighboring recipients. In the IoT, the SRAM memory works as a buffer when it receives and transmits data. Most controllers have a small SRAM size, for example, Arduino controllers SRAM, in comparison to many boards (shown in Table 1) [42].

**Figure 4:**
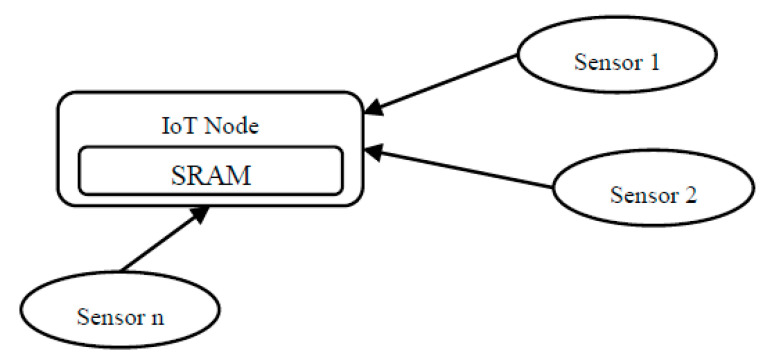
Multi sensors to one IoT node architecture.

To clarify the problem, Figure 4 illustrates how many sensors (from Sensor 1 to Sensor *n*, where *n* is an undetermined number) try to send their data to the SRAM memory of a connected IoT node, and sometimes the sensors send the data simultaneously and cause overflowing the IoT SRAM. Hence potential problems here are memory overflow and the possible loss of data due to buffer overflow. The probability of these problems increases, especially when more sensors are connected to the IoT node.

(1) $$total\;messages\;in\;one\;millisecond=\overset{n}{\underset{i=0}{\sum }}S_{n}.DF$$

where *S* denotes the sensor, *i* the number of sensors, which ranges from 1 to *n*, where *n* is the sensor’s max count connected to an IoT node. *DF* is the data flow from the sensor to the IoT node. If we have at least 2 bytes every millisecond, we can calculate the data flow size for one second from the following example: If the total number of messages sent in one second from sensor 1 = 2 Bytes × 1000 = 2000 Bytes ~ 2 KB/1 second, 2 KB is the max capacity of the IoT memory (SRAM). It has been found that the size of the transmitted data from all sensors can collapse the IoT node memory. To solve this problem, many solutions were proposed, such as limiting the count of sensors connected to the IoT node, adjusting the time interval in order to control when the sensor sends the data—i.e., when the controller reads sensor data—although, the fact remains that less read means less accuracy, or adjusting the packet size, sent from the sensor to the IoT node, which is not reliable to send fewer numbers. For example, instead of sending the integer 25, send 2, then 5, or just 2. Therefore, the best solution is to compress the data immediately when received using a compression algorithm suitable to work within the IoT memory limits and processer power. In the next section, we will investigate data compression algorithms.

### 2.3. The IoT Data Traffic Reduction Motivations

After collecting the data from sensors inside IoT memories, every node sends its data packets to the servers through IoT gateways, as illustrated in the IoT architecture in Figure 2. Thus, the number of sensors and IoT nodes directly affects the size of the data transmitted to the server. However, there are limitations for any network system, such as connection bandwidth, which could overflow when trying to send massive data in a period that the bandwidth of the network cannot handle. Furthermore, connection overflow could occur when sending an abundance of connection requests from clients to the server during a period that cannot be handled by the server, thus pushing the server to drop many of these connections. As a solution to these problems, compressing the data during the first stages before sending it to the servers will minimize connection sessions and reduce data traffic. Compression means that instead of sending the original data, we can send data of a smaller size, which will consume less battery and need fewer connection sessions and less time. For example, if the original data was 100 MB and the network bandwidth was 10 MB/S, it would take 10 turns to send this data, where every turn takes a second, which needs 10 s for sending the entirety of the data. However, if this data was compressed to 10 MB, the time needed would be reduced to one second, which reduces the network use by about 90%. Accordingly, this reduces data traffic and makes bandwidth available for service and for transmitting other data.

### 2.4. The IoT Data Compression State of Art

Many studies on aggregation and compression have been conducted in WSNs as the backbone of IoT networks [43,44], however, they mostly used compression at the servers because these nodes have more processing power than the sense/edge nodes and they do not have consumption problems, and that did not reduce much the traffic [45]. On the other hand, a IoT network differs from a WSN in terms of connectivity between each node, whereby the IoT node can be connected directly to the internet and has the ability to make decisions [46,47]. Therefore, a new way of aggregation and compression became in demand in IoT edge and sense nodes as the number of connected IoT devices and data increased exponentially during the last years [48,49].

Therefore, to deal with such large IoT data, a method was proposed as an update and a query-efficient index system in [4,50], with several criteria such as regular and necessary multidimensional updating of data. Some researchers stated that traditional database systems are not capable of handling large volumes of data and cannot support millions of data inputs per minute [51]. Other researchers in [52] stated that it could be highly impossible to move enormous data from IoT peripheral nodes to the server in a timely fashion and they stated that IoT devices should be able to store data, process, analyze, and sometimes make decisions in real time. Despite the IoT’s memory limitations, many machine intelligent algorithms have been proposed in [53] (ASIC-based acceleration [54], FPGA-based acceleration [55], mobile SoC-based acceleration [53]) in order to accelerate convolutional neural networks (CNNs) on embedded platforms. They focused on accelerating processing [56] and decreasing its energy consumption [57,58]. Few researchers have focused on data compression to minimize data size by retaining identical information content [36]. Although they have proposed that different algorithms compress data, because of various factors, the performances of these algorithms differ. These include factors such as power consumption [13], speed of data transmission [59], bandwidth [60], size of transmitted data [61], and processor power [62]. All these factors affect the IoT network’s performance directly.

The motivation to use compression algorithms comes from the small memory capacity of IoT devices, which works either as a buffer or cache memory in IoT networks, as researchers have stated in [63]. Some researchers in [31] have suggested data compression as a technique to reduce data traffic in the network and to empower IoT capability while others focused on power consumption; for example, Kimura and Latifi in [64] stated that energy consumption of one bit transmission via radio is 480 times higher than conducting one addition process. Some researchers tried to classify compression algorithms depending on the type of data, for example, algorithms that rely on the temporal correlation of sequenced residue data, as shown in [44,65], where they used information for compression like in [66]; therefore, they proposed S-LZW, SHuffman, and ND-encoding algorithms as examples. Another type of algorithm depends on data prediction [67], which has been considered more complicated and has several drawbacks such as high power consumption and large memory requirements, which is not available in most IoTs; for example, the MinDiff algorithm in [66]. Many data compression algorithms were proposed, such as coding by ordering, which dropped some sensor nodes and their data in an aggregated node [68]. Another method is pipelined in-network compression, which has been proposed for audio and video sensors and depends on the common similarity of data packets in bit values in order to delete the redundancies in data packets. Yet another method was proposed as a low-complexity video compression algorithm in [55] for video surveillance sequences collected by a wireless sensor network, where researchers introduced a framework based on change detection and JPEG compression of the region of interest (ROI); they stated that the proposed compression algorithm is similar to MPEG-2 and available at a much less computational cost. Another algorithm is distributed compression, which is used to obtain data from many spatial sources. The central node compares every sensor partial data with the data from the reference node in order to determine if there are any changes or errors, then decides what to send over the network and how to remove spatial redundancy [69,70]. Although some of these algorithms have been applied on WSNs, none of them have been applied for the IoT. The next section investigates compression techniques in order to determine which could better fit in IoT networks.

## 3. Compression

Compression is a way to represent massive data, which could be numeric, text, pictures, videos, and audios, or any other type, using a small data size. Compression is categorized into lossy and lossless. Lossy means the decompressed data is different from the original one while lossless compression is identical to the original and the decompressed data. The selection from the two types of compression techniques or algorithms depends on the type of data to be compressed. For example, to compress a picture using lossy compression, one should only keep enough information to know what is inside the picture, such as a car or a person. In contrast, lossless compression is not suitable for sensitive data such as financial or election data where it is used to alleviate transmission on the internet or storing data on USB drives. Therefore, when every single bit of data is critical, lossless data compression is used; otherwise, lossy compression is used. For video, audio, and picture data, it is better to use lossy compression because the accuracy and the compression ratio are high, otherwise, the original files are too large to be transmitted. For text and numerals or symbols, it is better to use lossless compression because identical data is required when decompressing. For example, we cannot rely on two words to replace ten words when representing the names of students, nor can we rely on two numbers to represent ten numbers because we will lose accuracy and sometimes transmit wrong data, which will lead to destructive results. However, IoT data only has numeric and text data format; therefore, using lossless data compression is the best solution.

### 3.1. Lossless Data Compression

A high compression ratio for any algorithm does not imply it is the best algorithm for all data types. Every data type has many suitable compression methods and algorithms. Many factors affect choosing the best compression method for every data type. However, it is known that the most influential compression factors are the speed of compression/decompression and compression ratio. Also, real-time data vs. offline data influences the selection of the compression algorithm as well. However, this paper focuses on lossless algorithms that have been proposed to compress numeric and time series data because the purpose of this paper is to investigate compression algorithms for IoT data. Therefore, three lossless compression types of algorithms were reviewed, which are categorized as entropy, dictionary, and general-based algorithms.

#### 3.1.1. Lossless Entropy Algorithms

Entropy encoding is a lossless data compression scheme in information theory, regardless of the medium’s specific characteristics. One of the main entropy coding types creates and assigns every single symbol of the entry into a unique prefix-free code. There are more than 16 algorithms support entropy algorithms such as Arithmetic Coding [71,72,73,74], Asymmetric Numeral Systems (ANS) [75,76,77], Golomb Coding [78,79], Adaptive Huffman [80,81,82], Canonical Huffman [83], Modified Huffman [84], Range encoding [85,86], Shannon [87], Shannon–Fano [88,89,90], Shannon–Fano–Elias [91], Tunstall coding [92,93], Unary coding [94,95,96], Universal Exp-Golomb [97,98], Universal Fibonacci Coding [99,100,101], Universal Gamma Coding [102,103], Universal Levenshtein Coding [104].

The main concept of entropy is to replace the symbol with a prefix code, which reserves a smaller size in the memory. In most of these algorithms, there is a need to store the symbols with their frequencies, which is then used in order to determine the replacement codes for the symbol, and this needs an abundance of memory. Furthermore, due to the complexity of searching and counting for the matched symbols and the encoding process itself, algorithms use more memory and need a large processing power that is not available in IoT devices; therefore, without modifying these algorithms, none of them would be suitable or applicable for the IoT systems and cannot be implemented on IoT nodes. The most potential candidate algorithm to be used after modification is the Adaptive Huffman because it can process real time inputs which is similar to the case of IoT inputs.

#### 3.1.2. Lossless Dictionary Based Algorithms

A dictionary-based algorithm is a scheme that creates a dictionary containing the symbols and the codewords assigned to it. The symbols are collected from the input data with no redundancy and represent all the input data, and the codeword assigned to every symbol should be smaller than the symbol itself, otherwise, inflation could happen. Many applications and algorithms create the dictionary dynamically, hence, when there is an input, the dictionary can be updated as needed. There are more than 19 algorithms support dictionary-based algorithms such as Byte pair encoding [105], Lz77 [87,106,107], Lz78 [74], (LZW) Lempel–Ziv–Welch [108], (LZSS) Lempel–Ziv–Storer–Szymanski [103,109,110,111], (LZS) Lempel–Ziv–Stac [112], (LZO) Lempel–Ziv–Oberhumer [113,114], Snappy [115,116], Brotli [117,118], Deflate [119], Deflate64 [120], LZ4 [121,122,123], (LZFSE) Lempel–Ziv Finite State Entropy [124,125], (LZJB) Lempel Ziv Jeff Bonwick [108], (LZMA) Lempel-Ziv-Markov chain-Algorithm [108], (LZRW) Lempel–Ziv Ross Williams [108,121,126], LZWL [127,128], LZX [129].

Entropy scheme algorithms rely on giving an index value for each symbol with the rule that each entry in the dictionary should not be iterated and has a unique index value. The dictionary size increases every time we have a new entry, which makes it a critical issue because the max size of the dictionary is limited according to the size of memory. The sliding window comes as a solution, which limits the entries for every interval. Every value in the sliding window is compared with previous indexed values in the dictionary. Hence, if the size of the dictionary increases, the search process for match symbols can take a long time, and this can make the encoding process even slower. All these are considered as obstacles for running any of these algorithms on an IoT node because of its low processing power and low memory size. Many modifications are needed, such as reducing the slide window size and limiting the dictionary size, to fit the IoT node specifications.

#### 3.1.3. Lossless General Compression Algorithms

Lossless general compression algorithms are implemented by replacing symbols in the context with codes or numbers in order to refer to their counts or predictions in the data, or by differences between the values if the input data is made of integers. The methods of these algorithms come in many shapes and steps, such as prediction at first followed by arithmetic coding that can be involved in order to encode the data. Hence, in this scheme, no dictionary or slide window is used. There are more than 8 algorithms support Lossless general compression algorithms such as Burrows-Wheeler transform (BWT) Burrows-Wheeler transform [130], (CTW) Context tree weighting [131], Delta [132,133], (PPM) Prediction by partial matching [134,135], (DMC) Dynamic Markov compression [136,137], (MTF) Move-to-front transform [138], PAQ [139], RLE [140,141].

Lossless general compression algorithms are different from entropy and dictionary-based algorithms in that they do not use a sliding window or create a dictionary. This is clear, especially in the BWT, Delta, and RLE algorithms The results of these algorithms depend on the sequence of input data, which is not guaranteed when dealing with IoT data. Most of the others need a large memory that exceeds the limits of IoT nodes. Furthermore, there is the complexity of encoding processes such as PPM and DMC algorithms that use arithmetic coding as a step or PPM and PAQ that use context mixing in order to increase the prediction preciseness. Many symbols move to the header of the stack in MTF, exceeding the limits of IoT nodes as well as all the mentioned algorithms.

## 4. Deep Learning

Deep learning is an evolution of machine learning mainly consisting of neural networks that aims to automate systems for many applications. It consists of neurons arranged in layers. Deep learning become popular recently due to its ability to provide accurate solutions in many domain problems. It has neurons, weight, bias and activation functions which need to be adjusted to obtain the best solution.

### 4.1. Deep Learning Architectures

There are various variants of deep learning in neural network architectures that consist of a wide variety of neural network training strategies [142,143]. Deep learning is divided into unlabeled and labeled data according to the type of data under processing. Autoencoder (AE) architecture [144,145] and restricted Boltzmann machine (RBM) architecture [146], which have been proposed by the so called “Father of Deep Learning”, Geoff Hinton, are considered the best for unsupervised learning and unlabeled data [147].

Both the architectures are considered to belong to the feature-extractor family and are supposed to be suitable for pattern recognition. For any work that involves the processing of time-series data, it is better to use a recurrent net (RNN) [148]. Supervised learning architectures are used for labeled data, such as using recursive neural tensor net (RNTN) and RNN for sentiment analysis [149], parsing [150], and entity/object recognition [151]. Deep belief networks (DBN) [152,153] and CNN [154,155] are used for images, objects [156], and speech recognition. RNN is used for speech recognition [157,158], entity recognition [159], and time-series analysis [160]. Many of the current deep learning architectures use one or a combination of previous solutions, depending on the data type they are analyzing.

Researchers in [161] stated that some functions have a complexity that cannot be handled in IoT devices without machine learning or deep learning. Other researchers in [162] explained that the obstacles of low memory and low processing power were the reason behind this. Despite this, the IoT and sensors’ data are the most common potential uses for brontobyte-level storage that is equal to 10 to the 27th power of bytes, as stated in [163]. Therefore, many scientists have studied how to reduce data traffic in order to alleviate the load on memory, as stated in [164,165]. The next paragraph illustrates the techniques used in deep learning in order to reduce the weights and number of parameters. These techniques are defined under dimensionality reduction, which represents big data using small, meaningful data by reducing its space [166]. Pruning and pooling are illustrated in more details to see if they can be used to reduce the data traffic.

### 4.2. Dimensionality Reduction Techniques

#### 4.2.1. Pruning

Pruning is a method used for various applications and areas. It is very commonly used in different ways to minimize complexity [146]. For example, it is used for mining spatial high utility co-location patterns based on actually shared weights and features [167]. However, pruning aims to make it fast and small in the neural network by reducing learning weights [168]. After training the network for the first time, all connections with weights below a threshold are deleted from the network. This process occurs whenever the network is retrained. The training results can minimize the network size by keeping sparse connections and neurons [169]. In [60] researchers used pruning and other techniques in order to compress neural networks. from the ImageNet ILSVRC-2012 dataset, researchers experimented on AlexNet Caffe to get 89% of weights pruned with 9× compression ratio and on VGGNet-16 to get 92.5% of weights pruned with 13× compression ratio. Researchers experimented on the MINIST dataset with two architectures. First, the Lenet-300-100, a fully connected network with two hidden layers, has 300 and 100 neurons in each layer. The second is the Lenet-5, which has two convolutional layers and two fully connected layers, they got 92% of weights pruned with 12× compression ratio for both architectures.

The ImageNet datasets describes the layer of convolutional (Conv) and full connected (Fc), while the MINIST datasets uses the layer of Conv and learnable parameters (lp). Each nodes describes the weights number and percent of weights pruned. The effectiveness of the pruning process was assessed in reducing the number of parameters and connections. Pruning removes the low-value weights and only keeps the high-value ones.

#### 4.2.2. Pooling

The pooling layer is used to reduce the features or the spatial volume of inputs. Pooling is usually used after the convolution layer or between two convolution layers [170]. The size of the dimension after pooling is reduced [155]. There are three types of pooling: minimum, average, and maximum pooling. CNN used pruning after convolution and before using a classifier to reduce complexity and avoid overfitting. This depends on dividing the convolved layer into disjoined regions, then determining the max or min or the average value for every region’s features [171,172].

## 5. Deep Learning Solutions for IoT Data Compression

Han in [60] proposed a deep learning algorithm to reduce the storage and energy required to run inference on large networks and deploy on mobile devices in three phases. He used pruning to reduce redundant connections, then applied quantization on weights to produce fewer codebooks that needed to be stored because many of the connections share the same weight. After that, Huffman coding was applied to effective weights. Although the experiment was not applied for IoTs, the results were promising. However, researchers in [173] tried to compress neural network structures into smaller matrices by finding the non-redundant elements. Other researchers in [174] proposed SparseSep for deep learning in order to fully connect layers for sparsification and for the separation of convolutional kernels in order to reduce the resource requirements. The authors in [175] stated that the large model’s group could be transferred to one small model after training using distillation, and that this would be much better for deployment. However, in [176,177], researchers proposed a dynamic network surgery compression algorithm to reduce the complexity of the network using the on-the-fly pruning method. They limited pruning in order to save accuracy. Therefore, they used the splicing method to compensate the important connections and weights that were pruned. Researchers in [178] worked on reducing the test time for the large convolutional network, which was directed for object recognition, starting with each convolution layer compressing and identifying the perfect low rank approximation before adjusting the top layers until the performance of the prediction was restored. Researchers in [179] investigated techniques for reducing complexity. Others tried to accelerate training by computing convolutions in the Fourier domain while reusing the same transformed feature map many times [180]. However, it is stated that most of the parameter values predicted need not be learned; architectures can be trained by learning a small weight number and predicting the others [181]. In order to improve model discrimination in responsive fields for local patches, a new network structure called “network in network” was suggested. It is a micro neural network that is instantiated with a multi-layer perceptron. This micro neural network is slid over the input in the same manner as CNN to generate the feature maps and use average pooling for classification [182,183]. Other researchers tried using information theory ideas in order to determine the optimal neural network size by having a tradeoff between complexity and a training error using second derivative information, which includes removing unimportant weights [184]. Researchers in [185] proposed a new method to train binarized neural networks at run-time; during forward propagation, this method greatly reduces the required memory size and replaces most operations with bit-wise operations [186]. However, binary weights were also proposed in [187], where researchers tried to replace the simple accumulations of several multiply-accumulate operations because multipliers took up most of the space and are considered power-hungry components when digital neural network is implemented. Another way to compress neural networks using a hashing trick was proposed in [188], where the idea of linking every group of weights in the same hash bucket with a single parameter using a hash function was proposed. The proposed method managed to minimize the model sizes significantly by exploiting redundancy in neural networks. Other researchers in [189] found that the use of k-means in weights clustering can lead to a very good balance between the size of the model and the accuracy of the recognition.

## 6. Experiments and Results

According to the specifications of the IoT data, this paper experiments on selected algorithms that need minimum memory, consume the least power, and have the potential to be modified and implemented into IoT nodes. The three algorithms that have been selected are Lz77 from sliding window algorithms, Lz78 from dictionary-based algorithms because these algorithms are considered to have the lowest complexity amongst the three, and the Huffman code from entropy algorithms, which been used in many compression applications and is very good for text compression with minimum complexity. Because the IoT data type can be heterogeneous since it comes from many different sensors, it is better to deal with this data as text instead of numbers. Otherwise, the data will have to be classified according to its sources, which will be more complex for the IoT device. The datasets used in the experiment are categorized into three types:

(1) The first type is a time-series dataset collected from sensors connected to IoT devices,
(2) The second type is time-series data not collected by sensors or IoT devices, and
(3) The third type is a collection of varied files, not time series, and not collected by sensors or IoT devices.

All three types of datasets were used in order to evaluate the performance of the proposed algorithms. All the experiments used at least 17 threads on a Dell server with a 2.4 GHz Intel Zeon 8 Cores E5620 46-bit-based processor and 100 GB RAM. Windows 10 Pro virtual was hosted on Centos 6, the operating system of the server. The five datasets with various dataset files are used for Compression Algorithms evaluation are: four data sets in the Dataset Kaggle [190], 5 in UCI database [191], 6 datasets in AMPDs [192,193], 10 datasets in The Calgary Corpus [194] and 6 datasets from Meteorology Department in Malaysian. The Malaysia Air Pollution consists 9 attributes: temperature, humidity, wind speed, wind direction, CO, O_3_, NO, NO_2_, and NOx for 6 stations: Cheras, Tanjong Malim, Putrajaya, Petaling Jaya and Nilai, Klang collected for 10 years between 01/01/2005 to 31/12/2016.

Compression algorithms were implemented on the previous datasets in order to evaluate these algorithms depending on the compression ratio that can be obtained by dividing the size of compressed files by the size of uncompressed. However, before calculating the compression ratio, the compressed size for each file should be calculated from the datasets according to every compression algorithm used. Table 2 shows the results for the dataset compression.

**Figure 5:**
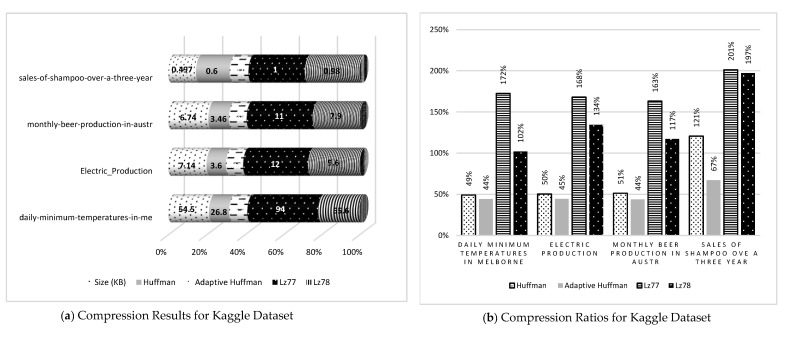

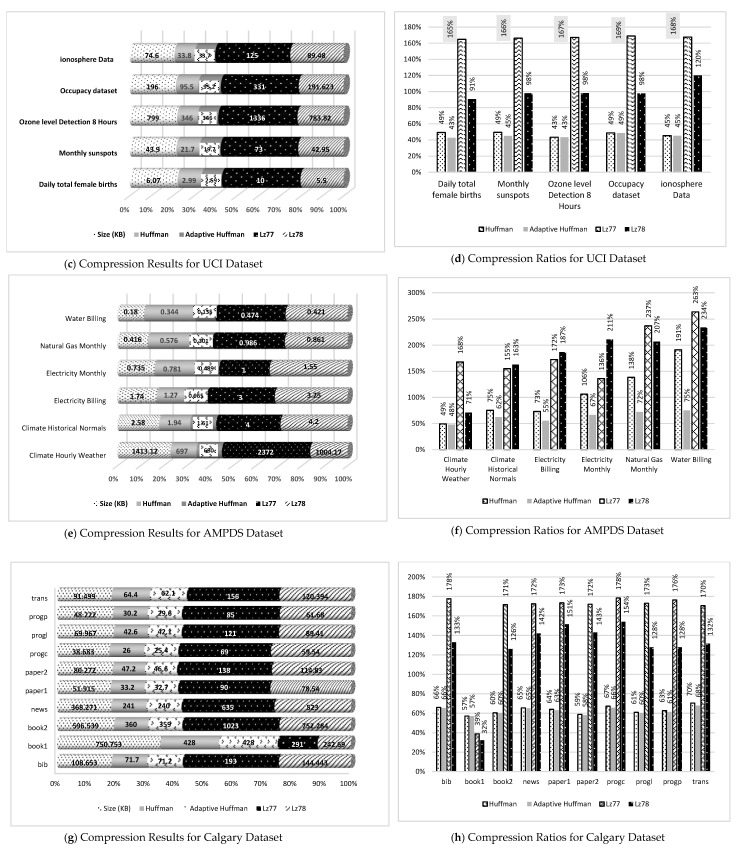

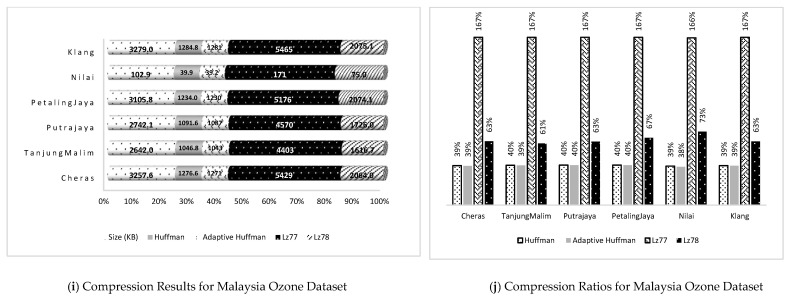
Compression results and ratios for all datasets.

Figure 5 shows the results and ratios of compression algorithms have been categorized by the source of the datasets. a, c, e, g, and i show the compression results, whereas b, d, f, h, and j show the compression ratios. It is clear from compression results that the adaptive Huffman algorithm had the best values in all the datasets, although it equaled the canonical Huffman in some results such as in ozone level detection for eight hours in c and Book1 in g. In contrast, Lz77 got the worst results—in some cases the sizes of compressed files were even bigger than the original ones in many cases because of an inflation problem. However, there were cases where Lz78 obtained the worst results, especially for electricity monthly, electricity billing, and climate historical normally in e, which proves that compression results depend on the distribution and iterations in data.

The compression results in a, c, e, g, and i show the comparison between compression algorithms when applying to the same files in datasets, whereas b, d, f, h, and j show the differences between compression ratios where the lowest compression ratio means better compression result. The adaptive Huffman also had the lowest compression ratio with one exception in h, where Lz78 got the lowest value for Book1 in the Calgary Corpus dataset.

Table 2 also shows the results categorized by data type; the minimum compression ratio is 32%, which resulted using Lz78 on Book1 from the Calgary Corpus dataset, where the maximum compression ratio is 263%, which resulted in using Lz77 on water billing data from the AMPDs dataset. However, for data type 1, the minimum compression ratio is 38%, which was obtained using adaptive Huffman, and for data type 2, the minimum ratio is 43%, which was also obtained using adaptive Huffman.

For data type 3, Lz78 is the lowest compression ratio when applying to Book1. However, if we exclude Book1 from the dataset, the adaptive Huffman would be the lowest ratio again, which is 58% ratio on paper2 from the Calgary Corpus. This means adaptive Huffman is the best when compressing time series and numeric data such as data type 1 and 2, however, it not necessarily good for data type 3.

The results clearly show that adaptive Huffman has a better compression ratio and is more significant than canonical Huffman. This means compressing real-time data is better than compressing offline data. On the other hand, Lz78, which is a dictionary-based algorithm, has more significant results than Lz77, which is a sliding window-based algorithm. However, some anomalies could happen, such as the three results in AMPDs dataset, where Lz77 has better compression ratios, and the reason for this was data sequence and redundancy as well as the file sizes, therefore the inflation problem can be noticed in the Lz77 sliding window in all the datasets.

## 7. Discussion

In the Compression section, it was found that not all the mentioned algorithms are suitable to be implemented in the IoT nodes without being modified because they require more memory and greater power processors than what an IoT node can provide. However, compression algorithms can be implemented in cloud servers or some aggregated nodes. These algorithms need a considerable space of stack and heap that should be reserved according to every algorithm code (arrays and pointers). Because of the differences between these codes, the size of the allocated memory could not be known before the implementation. Furthermore, the size of the data itself, in some cases, could require hours to be compressed.

The Deep Learning section explains that it is rather difficult to determine how many features are required to recognize an object, classify an image, or carry out other deep learning functions. These processes evolve deferent tasks according to the architecture used, and they also depend on the data type under processing. Therefore, every deep learning architecture has a different scenario. All architectures aim to know the minimum number of features in order to have the knowledge of which feature is good enough to have satisfied outputs with minimum errors. They transformed the high-dimensional data space into small-dimensional data space, which in turn conserves the same original data properties. High-dimensional data has many problems. It requires more time and space complexity and can also lead to overfitting. Furthermore, not all the features in high-dimensional data are involved or related to the problem we are solving. Reducing the dimension of data space leads to reducing the noise and unnecessary parts of data and helps to determine the features most related to the problem. Two approaches to apply dimensionality reduction were proposed. The first is feature selection, where the most related features to the problem are selected. The second is feature extraction, where new features from the high-dimensional data space are assessed to create the low-dimensional data space. Many deep learning techniques could be used for this, such as principal component analysis (PCA), non-negative matrix factorization (NMF), kernel PCA, graph-based kernel PCA, linear discriminant analysis (LDA), generalized discriminant analysis (GDA), Autoencoder, t-SNE, and UMAP. However, in order to avoid the problems or curses of dimensionality, the K-nearest neighbor algorithm (k-NN) is most commonly applied.

Traditional compression algorithms, as illustrated earlier in the Compression section, have a different meaning. In deep learning, compression in many architectures means minimizing the number of neurons or weights by removing them from layers, and this process is achieved by using the dimensionality reduction techniques. It is categorized as lossy compression, where lost information after compression does not fit the aim of IoT data compression. One of the first steps in deep learning architectures is initializing the values of the weights, which is done randomly, as illustrated in Figure 5. This process alone makes the output values unequal compared to the input data in the first layer, even though these output values could be very high accuracy. Furthermore, the process of deep learning is carried out in one direction from the input layer to the output layers. Activation functions are used through this process in order to determine which neuron values are relied upon to drop or keep these neurons and their connected weights. Hence, using activation functions breaks the linearity by retaining sparred values randomly and then training the model. When implementing the activation functions, the model starts from scratch with different weights values and leads to different results and outputs. However, previous results show some cases have a very close similarity with the original inputs and have smaller sizes and dimensions, as we have in lossy compression algorithms, which are acceptable in some cases and applications.

## 8. Conclusions, Challenges and Future Work

This paper reviewed smart cities’ issues and the importance of IoT in reducing data traffic, especially between sensors and IoT nodes. The current compression algorithms have limitations when trying to implement them using the IoT’s small memory. Lossy compression algorithms are not suitable due to the loss of information after transmission. In contrast, applying lossless compression algorithms is complex for IoT devices. Deep learning using pruning and pooling methods was applied in order to reduce data. However, it uses a lossy approach and does not aim for connections between sensors and IoT devices. In the future, a new algorithm using deep learning techniques combined with the lowest complex lossless compression algorithm and has the best compression ratio is needed. The suggested algorithm should fit the sensors and IoT data type and aim to produce a good compression ratio on every IoT node that reduces the network data traffic and transmits data faster, has higher utilization, and has better throughput.

## Figures and Tables

**Table 1:** Controllers Comparison Board Specs.

Name	Processor	Operating/Input Voltage	CPU Speed	EEPROM [KB]	SRAM [KB]	Flash [KB]
101	Intel^®^ Curie	3.3 V/7–12 V	32 MHz	-	24	196
Gemma	ATtiny85	3.3 V/4–16 V	8 MHz	0.5	0.5	8
LilyPad	ATmega168V	2.7–5.5 V/	8 MHz	0.512	1	16
	ATmega328P	2.7–5.5 V				
LilyPad SimpleSnap	ATmega328P	2.7–5.5 V/2.7–5.5 V	8 MHz	1	2	32
LilyPad USB	ATmega32U4	3.3 V/3.8–5 V	8 MHz	1	2.5	32
Mega 2560	ATmega2560	5 V/7–12 V	16 MHz	4	8	256
Micro	ATmega32U4	5 V/7–12 V	16 MHz	1	2.5	32
MKR1000	SAMD21 Cortex-M0+	3.3 V/5 V	48 MHz	-	32	256
Pro	ATmega168	3.3 V/3.35–12 V	8 MHz	0.512	1	16
	ATmega328P	5 V/5–12 V	16 MHz	1	2	32
Pro Mini	ATmega328P	3.3 V/3.35–12 V	8 MHz	1	2	32
		5 V/5–12 V	16 MHz			
Uno	ATmega328P	5 V/7–12 V	16 MHz	1	2	32
Zero	ATSAMD21G18	3.3 V/7–12 V	48 MHz	-	32	256
Due	ATSAM3X8E	3.3 V/7–12 V	84 MHz	-	96	512
Esplora	ATmega32U4	5 V/7–12 V	16 MHz	1	2.5	32
Ethernet	ATmega328P	5 V/7–12 V	16 MHz	1	2	32
Leonardo	ATmega32U4	5 V/7–12 V	16 MHz	1	2.5	32
Mega ADK	ATmega2560	5 V/7–12 V	16 MHz	4	8	256
Mini	ATmega328P	5 V/7–9 V	16 MHz	1	2	32
Nano	ATmega168	5 V/7–9 V	16 MHz	0.512	1	16
	ATmega328P			1	2	32
Yùn	ATmega32U4	5 V	16 MHz	1	2.5	32
	AR9331 Linux		400 MHz		16 MB	64 MB
Arduino Robot	ATmega32u4	5 V	16 MHz	1 KB (ATmega32u4)/512 Kbit (I2C)	2.5 KB (ATmega32u4)	32 KB (ATmega32u4) of which 4 KB used by bootloader
MKRZero	SAMD21 Cortex-M0+ 32 bit low power ARM MCU	3.3 V	48 MHz	No	32 KB	256 KB

**Table 2:** The Compression Results for Applying Algorithms on Datasets.

Dataset	Type	File Name	Size	Huffman	Huffman Ratio (%)	Adaptive Huffman	Adaptive Huffman Ratio (%)	Lz77	Lz77 Ratio (%)	Lz78	Lz78 Ratio (%)
Kaggle	1	Daily-minimum-temperatures-in-me	54.500	26.800	49	24.200	44	94.000	172	55.600	102
Kaggle	1	Electric_Production	7.1400	3.600	50	3.180	45	12.000	168	9.600	134
UCI	1	Monthly sunspots	43.900	21.700	49	19.700	45	73.000	166	42.950	98
UCI	1	Ozone level Detection 8 Hours	799.000	346.000	43	346.000	43	1336.000	167	783.820	98
UCI	1	Occupancy dataset	196.000	95.500	49	95.200	49	331.000	169	191.623	98
UCI	1	Ionosphere data	74.600	33.800	45	33.700	45	125.000	168	89.480	120
AMPDS	1	Climate hourly weather	1413.120	697.000	49	680.000	48	2372.000	168	1004.170	71
AMPDS	1	Climate historical normals	2.580	1.940	75	1.610	62	4.000	155	4.200	163
AMPDS	1	Electricity monthly	0.735	0.781	106	0.489	67	1.000	136	1.550	211
AMPDS	1	Natural gas monthly	0.416	0.576	138	0.301	72	0.986	237	0.861	207
Ozone	1	Cheras	3257.585	1276.561	39	1271.000	39	5429.000	167	2063.960	63
Ozone	1	TanjungMalim	2641.970	1046.768	40	1043.000	39	4403.000	167	1616.730	61
Ozone	1	Putrajaya	2742.100	1091.609	40	1087.000	40	4570.000	167	1726.020	63
Ozone	1	PetalingJaya	3105.790	1234.014	40	1230.000	40	5176.000	167	2074.050	67
Ozone	1	Nilai	102.932	39.881	39	39.200	38	171.000	166	75.000	73
Ozone	1	Klang	3279.01	1284.780	39	1281.000	39	5465.000	167	2075.070	63
Kaggle	2	Monthly beer production in Australia	6.740	3.460	51	2.950	44	11.000	163	7.900	117
Kaggle	2	Sales of shampoo over a three year period	0.497	0.600	121	0.334	67	1.000	201	0.980	197
UCI	2	Daily total female births	6.070	2.990	49	2.590	43	10.000	165	5.500	91
AMPDS	2	Electricity billing	1.740	1.270	73	0.965	55	3.000	172	3.250	187
AMPDS	2	Water billing	0.180	0.344	191	0.135	75	0.474	263	0.421	234
Corpus	3	bib	108.653	71.700	66	71.200	66	193.000	178	144.443	133
Corpus	3	book1	750.753	428.000	57	428.000	57	291.000	39	242.690	32
Corpus	3	book2	596.539	360.000	60	359.000	60	1023.000	171	752.284	126
Corpus	3	news	368.271	241.000	65	240.000	65	635.000	172	523.000	142
Corpus	3	paper1	51.915	33.200	64	32.700	63	90.000	173	78.540	151
Corpus	3	paper2	80.272	47.200	59	46.600	58	138.000	172	114.830	143
Corpus	3	progc	38.683	26.000	67	25.400	66	69.000	178	59.540	154
Corpus	3	progl	69.967	42.600	61	42.100	60	121.000	173	89.410	128
Corpus	3	progp	48.222	30.200	63	29.600	61	85.000	176	61.680	128
Corpus	3	trans	91.499	64.400	70	62.100	68	156.000	170	120.394	132

## Data Availability

Relevant Data are available at [190,191,192,193,194].
